# Improving TB care services among coal mine workers and their associated communities in Pakistan

**DOI:** 10.5588/pha.25.0011

**Published:** 2025-09-03

**Authors:** K.U. Eman, G.N. Kazi, Z.Z. Qin, J. Creswell, S.A. Raisani, U.R. Lodhi, N.A. Vasquez, S. John

**Affiliations:** ^1^ Dopasi Foundation, Islamabad, Pakistan;; ^2^ Stop TB Partnership, Geneva, Switzerland;; ^3^ Provincial TB Control Program Balochistan, Quetta, Pakistan;; ^4^ Research Institute of the McGill University Health Centre, Montreal, QC, Canada;; ^5^ Jannah Health Foundation, Yola, Nigeria.

**Keywords:** tuberculosis, active case finding, coal miners, presumptive TB, bacteriologically confirmed TB, prevalence

## Abstract

**SETTING:**

Five major coal mining districts in Balochistan, Pakistan.

**OBJECTIVES:**

To assess burden of TB among coal miners and their associated communities and establish linkages with TB care services.

**DESIGN:**

A cross-sectional study was conducted to find TB cases through active case finding. The target population included people working at coal mining sites and surrounding communities residing within 10 km, including coal miners’ families and other individuals. Verbal symptom screening was carried out via mobile camps and community outreach. Sputum was collected from screened positive individuals and tested for TB on GeneXpert. TB cases diagnosed were linked with TB care services.

**RESULTS:**

A total of 14,541 individuals including 8,149 (56%) coal miners were screened. Of the people screened, 81% were male, median age was 31 years, 2,274 (15.6%) had TB symptoms, and 34 confirmed TB cases were diagnosed. All 34 TB patients were linked to care and 32 completed treatments successfully. The estimated TB prevalence was 234 cases per 100,000 population (95% confidence interval: 150.6–316.5), with no significant difference between coal miners and associated communities.

**CONCLUSION:**

Similar TB prevalence among coal miners and associated communities reflects shared vulnerability. Use of more sensitive screening tools is recommended to validate prevalence estimates in future studies.

Every year, nearly 11 million people are affected by TB worldwide, mostly in Africa and Asia.^1,2^ Globally, TB exhibits significant disparities across regions and disproportionately affects vulnerable and marginalised populations.^3^ Given these disparities, early screening and diagnosis among high-risk populations are prioritised interventions in the End TB Strategy,^4^ the Global Plan to End TB,^5^ and the WHO targets for TB elimination by 2035,^6^ in alignment with the United Nations Sustainable Development Goal 3.

Pakistan ranks fifth among high-TB-burden countries, with an estimated 686,000 people developing TB in 2023. Nearly 30% of these cases are estimated to have been missed by the National TB Program (NTP).^1^ TB has consistently remained the topmost cause of death from infectious diseases in Pakistan since 1990.^7^ Although Pakistan’s NTP guidelines emphasise prioritising high-risk groups in the TB response, identifying and effectively engaging these populations remains a significant challenge.^8^

People working in mines have since long been recognised as a key population at risk for TB.^9^ Several studies from sub-Saharan Africa have reported a high prevalence of TB among mineral mine workers due to exposure to silica and poor ventilation.^10–13^ Most of these studies have focused on gold mining. Miners in southern Africa are reported to have TB incident rates up to 10 times higher than the general population and four-fold higher risk of death from TB.^10,11^ In Zambia, 9.5% prevalence of active pulmonary TB has been reported among copper mine workers.^12^ Mining is also a risk factor for other lung conditions, which have not been studied as extensively.^14^ Communities living near mining sites also face increased vulnerability to respiratory conditions such as pulmonary fibrosis, silicosis, and TB due to their proximity to toxic particles produced during mining activities.^14^ However, these groups often remain underrepresented in health care networks due to their low social visibility.

In Pakistan, the coal mine industry has traditionally entailed hazardous working conditions due to insecure infrastructure, congested environments, and dangerous underground operations, injurious to the physical well-being of workers.^15,16^ In the Sindh province, over 25% of Lakhra coal miners experienced persistent coughing and breathing problems.^17^ The Balochistan province accounts for most of Pakistan’s total coal production, with mines located in remote mountainous terrains, over 40 km from major cities.^18,19^ Overall, the province has the highest proportion of TB cases missed (67%), owing to low population density and highly scattered population clusters. Thus, while the resource-constrained coal mining communities face multiple health issues, they have almost no access to health care facilities including TB services.^19^

Active case finding (ACF) is recommended by the WHO for high-TB-prevalence groups to systematically screen individuals who do not regularly visit health care facilities.^2^ Various ACF strategies have proven effective in identifying people with TB at earlier stages, and their context-specific nature can help reduce TB incidence through early diagnosis, enable prompt care, and shorten the duration of illness.^20^ We implemented an ACF intervention aimed at screening coal miners and their associated communities to estimate burden of TB and link individuals with TB to appropriate treatment and care through the public health care system.

## METHODS

**FIGURE 1.**
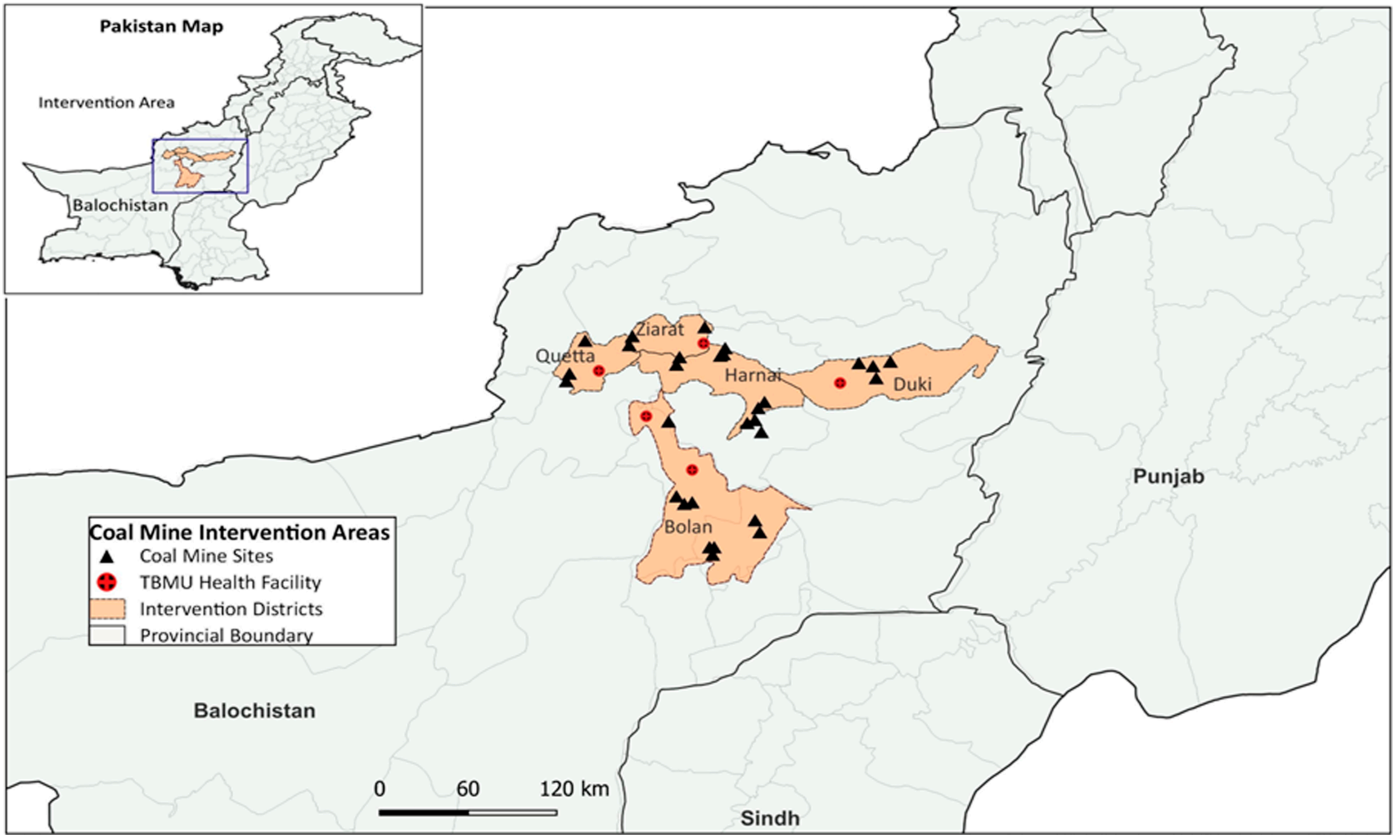
Active case finding intervention in major coal mining districts of Balochistan.

The intervention was implemented in five major coal mining districts of the Balochistan province: Quetta, Bolan, Harnai, Dukki, and Ziarat (Figure 1). These districts were selected based on the abundance of coal deposits and their role in employing approximately 80% of all coal miners, the majority of whom are migrants from other provinces.^21,22^ According to the Provincial Mines and Minerals Department, these districts contain approximately 840 functional coal mines, each employing an average of 15 workers.^21^ However, the actual number of coal mines in Balochistan is likely much higher due to the presence of unregistered operations. Estimates from grey literature suggest that between 150,000 and 200,000 miners may be working in the province.

The study target population included coal miners and their associated communities. Coal miners included all people working at selected coal mining sites regardless of the nature of the work. Associated communities included people residing within 10 km distance of selected coal mines, including families of the coal miners, contacts of TB cases, and individuals interacting directly with miners like transporters and drivers. Individuals of both sexes aged 10 years and above were eligible to participate.

### Mapping and site selection for intervention

Before initiating the ACF activities, a mapping exercise was conducted to gather data on mining sites and nearby health facilities to facilitate the selection of screening camp locations. Mobile screening camps were held at locations within 1 km radius of functional mines in areas where 50 or more individuals were estimated to be available for screening, following prior permission from the mine owners.

Community outreach activities were implemented within 10 km of the selected mines.

### Community mobilisation

Prior to each screening camp, a community gathering was organised at coalmining sites to raise awareness and mobilise local support. Information on TB symptoms, treatment, and prevention was provided with the help of brochures. These gatherings, attended by community leaders, coal mine owners, and district TB coordinators, encouraged miners to participate in the screening without fear of stigma or job loss, and also helped identify accessible locations for the screening activities.

### Intervention and recruitment

The project implemented three ACF screening strategies: 1) camps for coal mine workers, 2) community outreach through door-to-door screening, and 3) contact tracing to screen individuals who had been in contact with people diagnosed with TB. Verbal symptom screening was used to identify presumptive TB. The symptom screening questionnaire included WHO-recommended four symptoms for TB: unexplained cough of more than two weeks, fever, weight loss, and night sweats.^23^ Participants having any of these symptoms were identified as presumptive TB, and a spot sputum sample was collected and transported to the nearest public sector laboratory for Xpert MTB/RIF (Xpert) testing. People diagnosed with TB were registered at the nearest TB Management Unit (TBMU) for treatment, in accordance with the NTP guidelines. All persons with TB were followed up till completion of the treatment, either through phone calls or by contacting the linked TBMUs.

Screening camps were held by field staff trained in verbal screening, sample collection, and data recording. House-to-house screenings in surrounding localities and contact screening were conducted, using the same verbal symptom screening approach.

### Data collection and analysis

After obtaining informed consent, each participant was assigned a unique study code, and a paper-based questionnaire was administered to screen for symptoms and collect basic demographic information. Contact details, including residential address, and date and time of sputum sample collection were also recorded for TB presumptive at the time of sample submission. The results of Xpert testing were documented, when available. Individuals who tested positive were informed of their results and linked to the nearest TBMUs. Final treatment outcomes were collected from respective TBMUs. The paper-based data collected were entered centrally into MS Excel spreadsheets. Stata 13.1 was used for analysis. The proportions of individuals progressing through the TB care cascade, including those screened for symptoms, identified as presumptive TB, tested in the laboratory, diagnosed with TB, initiated on treatment, and those who completed treatment were analysed. Chi-square tests were performed to assess potential differences between population subgroups, such as coal miners versus associated communities and female versus male. Based on the number of people screened and TB cases diagnosed, crude TB prevalence was estimated for 100,000 population.

### Ethics statement

All activities were implemented in accordance with the National TB Guidelines for screening and ACF among high-risk populations. This study was conducted in accordance with the ethical principles, participants were informed about the intervention, and verbal informed consent was obtained. All data were anonymised to ensure confidentiality and privacy. All results presented in this study are derived directly from programmatic intervention data.

## RESULTS

Between January and December 2019, a total of 96 screening camps were conducted at selected mining sites. A total of 14,541 individuals were screened, including 8,502 (56%) coal miners and 5,967 (44%) community members through outreach activities, and 72 individuals through contact tracing. Participants had a median age of 31 years, with an interquartile range of 14. Women accounted for 19% (n = 2763) of all individuals screened, including only 48 (0.6%) among coal miners and 2,715 (42%) among community members (Table).

**TABLE.** TB screening, diagnosis, prevalence, and treatment outcomes among coal miners and associated communities.

	Total, N (%)	Coal miners, N (%)			Associated communities, N (%)			*P* value*
		Men	Women	Total	Men	Women	Total	
People screened	14,541	8,101	48	8,149	3,677	2,715	6,392	
People with presumptive TB	2,274 (15.6)	1,356 (16.7)	27 (56.3)	1,383 (16.9)	534 (14.5)	357 (13.1)	891 (13.9)	<0.001
People positive for TB	34	17	2	19	5	10	15	
Proportion of TB positive among presumptive	(1.5)	(1.3)	(7.4)	(1.4)	(0.9)	(2.8)	(1.7)	0.553
Proportion of TB positive among screened	(0.23)	(0.21)	(4.17)	(0.23)	(0.14)	(0.37)	(0.23)	0.985
Number needed to								
Screen to find one presumptive TB	6.4	6.0	1.8	5.9	6.9	7.6	7.2	
Test to diagnose one TB case	67	80	14	73	107	36	59	
Treatment outcomes								
Patients initiated on treatment	34	17	2	19	5	10	15	NA
Patients successfully treated	32	16^**A**^	2	18	5	9^**A**^	14	<0.001
Crude estimate of TB prevalence/100 K	234	210	4,167	233	136	368	235	0.98

*P values for the differences between coal miners and associated community members.

A One male Afghan migrant lost to follow-up due to out-migration, and one woman died due to comorbidities.

Among individuals screened, 2,274 (15.6%) reported symptoms suggestive of TB, with significantly higher rates (*P* ≤ 0.001) among coal miners (17%) compared with community members (13.9%). A similar trend was observed across both sexes (Table). Overall, the proportion of individuals with presumptive TB was higher (*P* = 0.005) among men (1,890/11,778 = 16.0%, 95% confidence interval (CI): 15.4–16.7) than women (384/2,763 = 13.9%, 95% CI: 12.6–15.2). A high proportion of presumptive TB was identified among the small number of female coal miners (27/48 = 56%), with minimal impact on the overall proportion.

**FIGURE 2.**
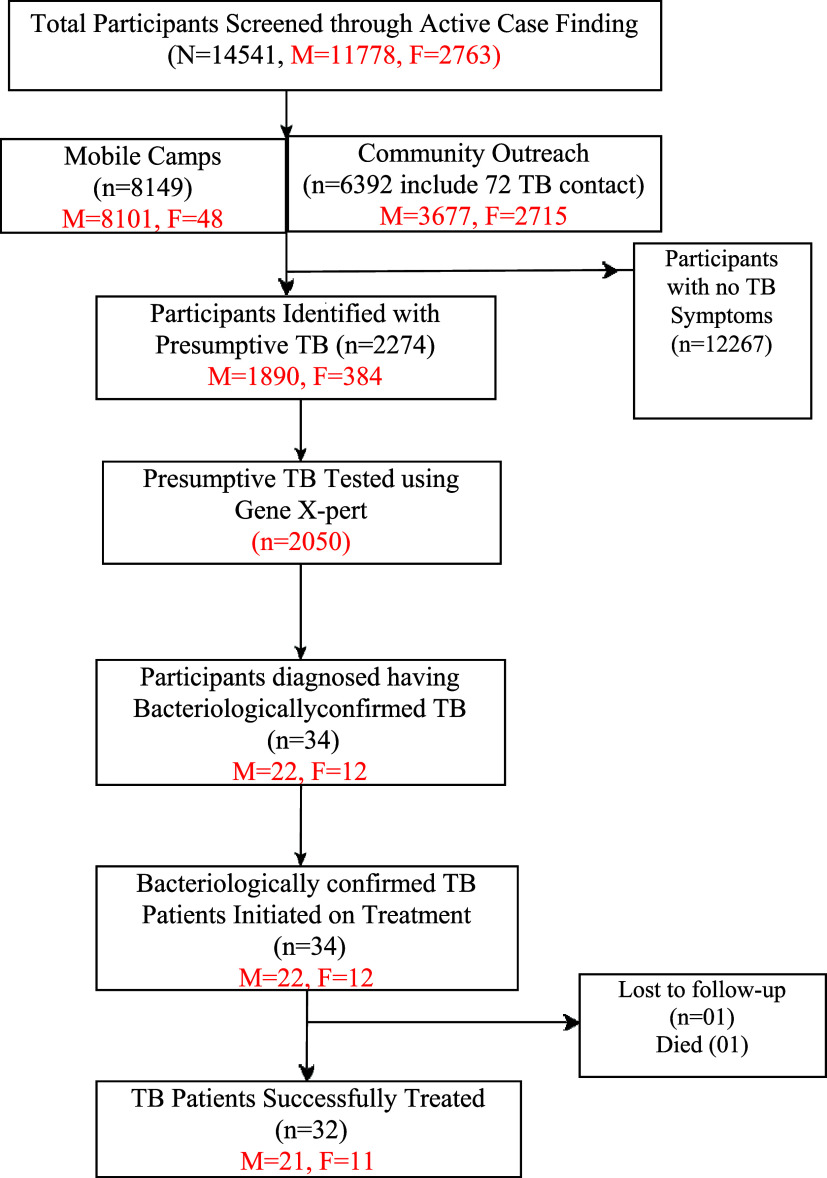
Flow diagram presenting individuals progressing through the TB care cascade from active screening, diagnosis, and TB treatment care.

Of presumptive TB, 2,050 (90%) individuals were able to produce sputum and 34 TB cases were diagnosed on Xpert MTB/RIF testing. The remaining 224 (9%), who could not expectorate sputum, were assessed clinically. All 34 TB cases diagnosed were initiated on treatment. Of these cases, 32 (94.1%) successfully completed treatment; one coal miner was lost to follow-up, and one female patient died due to comorbidities (Figure 2).

The proportion of TB cases among individuals with presumptive TB was not statistically different between coal miners and community members (*P* = 0.553). However, it was significantly higher in women (12/384 = 3.13%; 95% CI: 1.38–4.86) compared with men (22/1,890 = 1.16%; 95% CI: 0.68–1.65) (*P* = 0.004). Among the screened population, the difference in the proportion of TB cases diagnosed between coal miners (1.4%) and community members (1.7%) was not statistically significant. Accordingly, the estimated crude TB prevalence (234 per 100,000 population) was similar between coal miners and their surrounding communities. However, the proportion of TB-positive cases among those screened differed significantly by sex (*P* = 0.015), with a higher estimated TB prevalence in women (434 per 100,000) compared with men (187 per 100,000).

## DISCUSSION

The ACF intervention targeted coal miners and nearby communities in five districts of Balochistan, Pakistan, aiming to assess TB burden and address TB-related inequities in high-risk groups with limited health care access and fear of diagnosis due to stigma and job loss.^24,25^ We reported a TB prevalence of 234/100,000 in study population, which is close to the estimated prevalence of 280 for the Balochistan province (95% CI: 69–490) but lower than national estimates of 348 per 100,000.^26^ We found similar rates of TB among miners and their communities. In contrast, a similar study from Iran reported a two-fold higher relative risk of acquiring TB infection associated with silica exposure.^27^ Moreover, the prevalence of TB was reported up to 10 times higher among coal miners compared with the general population in South Africa and Malawi.^10,28,29^ Our lower study estimates might be attributed to healthy worker bias among the coal miners, as those with active TB symptoms may abstain due to illness or may not have consented to participate in the study. Further inquiries to identify factors for low TB diagnosis revealed that most coal miners hailed from other provinces/districts as has also been reported previously.^22^ Furthermore, it was informed that immigrant coal miners upon falling ill return to their homes mostly in the Shangla district of the Khyber Pakhtunkhwa province.

The low number of TB diagnoses in our high-risk population is likely attributable to the reliance on symptom-based screening alone. Evidence from prevalence surveys conducted in Asia indicates that 40%–79% of bacteriologically confirmed TB cases were asymptomatic and would have been missed in the absence of chest X-ray (CXR) screening.^30^ Using CXRs in ACF could have improved detection in our study population. Furthermore, the National TB Prevalence Survey conducted in Pakistan in 2010^26^ reported relatively lower rates of TB among individuals aged 15–34 years compared with older age groups, which may have influenced our study’s results. Coal miners and nearby communities are more prone to respiratory illnesses like pneumoconiosis and silicosis, which share symptoms with TB, potentially increasing presumptive TB cases.^14,31^ In our study, 15.6% had TB symptoms, with coal miners being more symptomatic than community members. However, only 1.5% of symptomatic individuals were diagnosed with TB, suggesting lung conditions other than TB. Addressing other lung conditions could improve TB response efforts.^32^ We found a similar prevalence of TB among associated communities in close proximity to mines compared with current coal miners, potentially attributable to exposure to mine dust and close contact with miners.^33^

The higher TB prevalence among women in our study aligns with trends in Balochistan and Afghanistan, where women make up a larger proportion of notified TB cases. Female miners often come from nearby communities, while male miners tend to come from other districts and return home when ill. Data on participants’ home districts or ethnicities were not collected, limiting the analysis. This underscores the need for targeted TB prevention and care for women in remote mining areas. While ACF can help identify cases early, ensuring equitable health care access, especially for rural women, remains a challenge. Further research is needed to explore the gender disparity and assess ACF’s impact on improving TB outcomes for women.

In our intervention, a similar proportion of community members was screened through outreach efforts and screening camps, underscoring the necessity of adopting a mixed or complementary approach in screening, tailored to each community’s unique socio-demographic and cultural characteristics and specific needs.^34^ It is essential to further evaluate the efficacy and cost-effectiveness of ACF interventions for coal miners and their communities, considering factors like camp accessibility, distance, and the acceptability of screening teams. These elements are key to tailoring programmes at national and provincial levels.

This intervention successfully linked people diagnosed with TB to TB care services, with the treatment success rate exceeding 94%. It shows that active TB case finding in vulnerable populations can efficiently lead to diagnosis and treatment initiation. ACF not only identifies more TB cases but also enables continued engagement during treatment, improving outcomes.^34^

The study had limitations, including the absence of CXR during ACF, which likely resulted in underreporting of TB cases, as symptom screening alone is known to miss many active cases. CXR was not used due to security concerns, emphasising the need for context-specific approaches in remote areas. Additionally, the lack of a sampling frame limits the generalisability of our findings.

## CONCLUSION

This study, conducted in remote coal mining areas, highlights the critical need to extend comprehensive health care services to both current coal miners and their surrounding communities, who face equally high TB burdens. Notably, the findings revealed disproportionately elevated TB rates among women in these communities, underscoring a key population requiring targeted interventions. While the observed TB prevalence was comparable to national levels, it was lower than the provincial estimates. This discrepancy warrants further investigation to explore the potential influence of healthy-worker bias effects and the implications of coal miners returning to their hometowns upon falling ill, particularly to the Shangla district in the Khyber Pakhtunkhwa province, from where 80% of these miners originate.
